# Low Levels of Serum Fetuin-A and Retinol-Binding Protein 4 Correlate with Lipoprotein Subfractions in Morbid Obese and Lean Non-Diabetic Subjects

**DOI:** 10.3390/life11090881

**Published:** 2021-08-27

**Authors:** Hajnalka Lőrincz, Imre Csige, Mariann Harangi, Anita Szentpéteri, Ildikó Seres, Zoltán Szabó, György Paragh, Sándor Somodi

**Affiliations:** 1 Department of Internal Medicine, Faculty of Medicine, University of Debrecen, 4032 Debrecen, Hungary; lorincz_hajnalka@belklinika.com (H.L.); harangi@belklinika.com (M.H.); szentpeteri_anita@belklinika.com (A.S.); seres@belklinika.com (I.S.); paragh@belklinika.com (G.P.); 2 Department of Emergency Medicine, Faculty of Medicine, University of Debrecen, 4032 Debrecen, Hungary; csige.imre@med.unideb.hu (I.C.); szabo.zoltan@med.unideb.hu (Z.S.); 3 Doctoral School of Health Sciences, University of Debrecen, 4032 Debrecen, Hungary

**Keywords:** fetuin-A, retinol-binding protein 4, lipoprotein, obesity, insulin resistance, diabetes

## Abstract

Background: Fetuin-A and retinol-binding protein 4 (RBP4) are secreted as both hepatokine and adipokine. These are involved in insulin resistance, obesity-related dyslipidemia, and atherosclerosis. To date, correlations of circulating fetuin-A and RBP4 with lipoprotein subfractions as well as high-density lipoprotein (HDL)-linked proteins have not been entirely investigated in morbid obese and lean non-diabetic subjects. Methods: One-hundred obese non-diabetic patients (body mass index, BMI: 42.5 ± 8.1 kg/m^2^) along with 32 gender and age-matched normal weight controls (BMI: 24.5 ± 2.5 kg/m^2^) were enrolled in our study. Serum fetuin-A and RBP4 were measured by ELISA. Lipoprotein subfractions were distributed by Lipoprint gelelectrophoresis. Results: Serum fetuin-A and RBP4 were unexpectedly lower in obese patients (*p* < 0.01 and *p* < 0.01, respectively) compared to controls and correlated with each other (r = 0.37; *p* < 0.001). Fetuin-A had positive correlations with HDL-C (r = 0.22; *p* = 0.02), apolipoprotein AI (apoAI) (r = 0.33; *p* < 0.001), very-low density lipoprotein (VLDL) subfraction (r = 0.18; *p* = 0.05), and large HDL subfraction levels (r = 0.3; *p* = 0.001) but did not show correlation with carbohydrate parameters in all subjects. RBP4 correlated positively with HDL-C (r = 0.2; *p* = 0.025), apoAI (r = 0.23; *p* = 0.01), VLDL subfraction (r = 0.37; *p* < 0.001), intermediate HDL subfraction (r = 0.23; *p* = 0.01), and small HDL subfraction (r = 0.21; *p* = 0.02) concentrations, as well as C-peptide levels in overall participants. Backward stepwise multiple regression analysis showed that serum fetuin-A concentration is best predicted by RBP4 and large HDL subfraction. In model 2, VLDL subfraction was the independent predictor of serum RBP4 level. Conclusions: Our data may indicate a potential role of fetuin-A and RBP4 in impaired lipoprotein metabolism associated with obesity.

## 1. Introduction

Incidence of central obesity and its related co-morbidities have been dramatically growing worldwide due to a sedentary lifestyle, fat and carbohydrate-rich nutrition, together with genetic effects. White adipose tissue and liver-generated hormone-like peptides—collectively termed as adipokines and hepatokines, respectively—have paracrine, endocrine, and systemic effects. The dysregulation of these adipo- and hepatokines (e.g., leptin, chemerin, plasminogen activator inhibitor-1, afamin, fetuin-A, retinol-binding protein 4 etc.) has been associated with central obesity and obesity-related co-morbidities including metabolic syndrome, type 2 diabetes, dyslipidemia, and enhanced atherogenesis. However, the downregulation of favorable adipokines, such as adiponectin, vaspin, and omentin-1, has been linked to insulin resistance [1]. Therefore, investigation of obesity-associated adipokine and hepatokine production has been intensively studied.

Fetuin-A or α-2-Heremans–Schmid-glycoprotein (α2-HS-glycoprotein, AHSG) is a multifactorial bioactive protein that is mostly produced by hepatocytes and partially by visceral adipose tissue. It is secreted into the circulation at relatively high concentration [2]. Being originally described as an essential developmental factor in the fetal circulation both in humans and animals [2], it serves as a potent inhibitor of vascular calcification in endothelial cells [3]. An increased level of fetuin-A is found to be involved in the development of insulin resistance through its effect on the endogenous inhibitor of insulin receptor tyrosine kinase [4]. Indeed, fetuin-A level is considered as a biomarker for obesity [5], dyslipidemia [6], and subclinical atherosclerosis [7]. In addition, a recent meta-analysis suggests that fetuin-A may prove to be an important indicator for the components of metabolic syndrome [8]. Genetic influences also impact fetuin-A concentrations and functions; AHSG single-nucleotide polymorphism was found to be susceptible for obesity and early-onset co-morbidities [9].

Similar to fetuin-A, retinol-binding protein 4 (RBP4) is synthetized and secreted as both hepatokine and adipokine. RBP4 transfers retinol (vitamin A) from the liver to the target tissues within the circulation in combination with transthyretin, which prevents renal filtration and the catabolism of RBP4 [10]. Although there has been an exponential growth of knowledge about the function of RBP4, data from physiological and pathological concentration of RBP4 are contradictory. Fan et al. described a U-shaped association between serum RBP4 concentrations and the risk of incident of type 2 diabetes in prediabetic patients [11]. Higher serum RBP4 levels were involved in obesity, type 2 diabetes, and in patients with cardiovascular disease [10,12]. A decreased GLUT4 expression in visceral adipose tissue also leads to an increased circulating concentration of RBP4 associated with insulin resistance in the hepatic and muscular cells [13]. Furthermore, current studies showed that RBP4 and retinoids are strongly involved in obesity-associated dyslipidemia [14], but results are not fully elucidated. 

Obesity-related dyslipidemia is one of the well-known complications in obese patients including higher concentration of very low-density lipoprotein (VLDL), small-sized low-density lipoprotein (LDL) subfractions, and decreased mean LDL size. In addition, we previously published a shift toward the small-sized high-density lipoprotein (HDL) subfraction in morbid obese patients compared to lean controls [15]. Recently, proteomic analyses on HDL described an extended list of HDL-associated proteins including fetuin-A, RBP4, and transthyretin [16]; however, some authors indicated that the data are highly controversial and strongly depend on HDL isolation as well as the purification method [17]. 

To date, data are highly contradictory about the direct association of fetuin-A and RBP4 molecule to HDL particle; moreover, data are lacking about the relationship between impaired circulating fetuin-A and RBP4 production and the markers of obesity-related dyslipidemia (i.e., small HDL, mean LDL size, etc.) in a special morbid obese but non-diabetic population. We hypothesized that there is a correlation between serum fetuin-A and RBP4 with lipoprotein subfractions, including HDL subfractions and other HDL-linked proteins in morbid obese, non-diabetic, and healthy lean participants.

## 2. Patients and Methods

### 2.1. Patients

We enrolled one-hundred non-diabetic obese patients from our obesity outpatient clinic at the Department of Medicine, Faculty of Medicine, University of Debrecen, Hungary. Thirty-two aged and gender-matched healthy lean volunteers (checked with routine laboratory parameters) were also enrolled from the university. Anthropometric and laboratory parameters of participants are summarized in Table 1. Permission to carry out this study was granted by the Regional Ethics Committee of the University of Debrecen and the Medical Research Council (registration number: DE RKEB/IKEB 5513B-2020 and IV/7989-1/2020/EKU, respectively). All participants signed a written informed consent. Obesity was defined as body mass index (BMI) ≥ 30 kg/m^2^. The main medications of obese patients are shown in Table 1. The control group was free of medication. Individuals with liver, kidney, endocrine disorders (including both type 1 and 2 diabetes mellitus), pulmonary, neurological, gastrointestinal, psychiatric, acute infective, autoimmune disease, or malignancies were excluded. Additional exclusion criteria including pregnancy, breast feeding, smoking, and regular alcohol consumption were applied.

### 2.2. Sample Collection and Biochemical Measurements

All venous blood samples were collected after a 12 h fasting. The routine laboratory parameters, including high-sensitivity C-reactive protein (hsCRP), fasting glucose, fructose amine, hemoglobin A1C (HbA1C), glomerular filtration rate (GFR), urea, creatinine, thyroid-stimulating hormone, liver enzymes, total cholesterol, triglyceride, HDL-cholesterol (HDL-C), LDL-cholesterol (LDL-C), apolipoprotein AI (apoAI), and apolipoprotein B (apoB) levels were determined from fresh sera with a Cobas c501 analyzer (Roche Ltd., Mannheim, Germany) from the same vendor. To check the carbohydrate parameters of healthy, control participants, fasting glucose and HbA1C levels were determined. A routine 75 g oral glucose tolerance test (OGTT) was applied after an overnight fast to check the non-diabetic status in obese participants. At the same time, fasting insulin and C-peptide levels were also measured in obese patients. Homeostasis model assessment–insulin resistance (HOMA-IR) was calculated with the formula of Matthews. Sera were kept frozen at −70 °C for subsequent enzyme-linked immunosorbent assay (ELISA) measurements and lipoprotein subfraction analysis.

### 2.3. ELISA Measurements

Circulating fetuin-A and RBP-4 concentrations were determined by ELISA (R&D Systems, Minneapolis, MN, USA) with 3.9–4.9% and 5.7–8.1% intra-assay and 7.4–8.4% and 5.8–8.6% inter-assay coefficients, respectively, according to the manufacturer’s recommendation.

### 2.4. Determination of PON1 Enzyme Activities

Serum PON1 arylesterase activity was measured with phenylacetate substrate (Sigma Aldrich, Budapest, Hungary), and the hydrolysis of phenylacetate was monitored at 270 nm at room temperature as previously described [15]. Serum PON1 paraoxonase activity was assayed on a microtiter plate by a kinetic, semiautomated method using paraoxon (O,Odiethyl-O-p-nitrophenyl phosphate, Sigma-Aldrich, Budapest, Hungary) as a substrate. Hydrolysis of paraoxon was followed at 405 nm at room temperature.

### 2.5. Lipoprotein Subfraction Analyses

LDL and HDL lipoprotein subfractions were distributed by the electrophoretic method on polyacrylamide gel with a Lipoprint System (Quantimetrix Corporation, Redondo Beach, CA, USA), as previously described [18]. Then, 25 µL serum was added to the polyacrylamide gel tubes along with 300 and 200 µL loading gel solution, respectively, containing Sudan Black as a lipophilic dye. The tubes were photopolymerized at room temperature for 30 min followed by electrophoretization at a constant of 3 mA/tube. Each electrophoresis chamber consisted of quality control provided by the manufacturer (Liposure Serum Lipoprotein Control, Quantimetrix Corporation, Redondo Beach, CA, USA). Subfraction bands were scanned with an ArtixScan M1 digital scanner (Microtek International Inc., CA, USA) and analyzed with Lipoware software (Quantimetrix Corporation, Redondo Beach, CA, USA).

In LDL subfraction analysis, up to seven LDL subfractions were distributed. The proportion of large LDL (large LDL %) was defined as the sum of the percentage of LDL1 and LDL2, whereas the proportion of small LDL (small-dense LDL %) was defined as the sum of LDL3-LDL7. Cholesterol concentrations of LDL subfractions were determined by multiplying the relative area under the curve (AUC) of subfractions by the total cholesterol concentration of the sample. Calculated total LDL-C is comprised of the sum of the cholesterol in Midbands C through A and LDL subfractions (LDL1-LDL7), and it strongly correlates with the directly measured LDL-C [19].

In HDL subfraction analysis, ten HDL subfractions were differentiated between LDL and albumin peaks. These were grouped into three major classes: large (from HDL1 to HDL3), intermediate (from HDL4 to HDL7), and small (HDL8 to HDL10) HDL subfractions. Cholesterol concentrations of the HDL particle subsets were calculated with Lipoware by multiplying the total HDL-C concentration of the samples by the relative AUC of the subfraction bands.

### 2.6. Statistical Analyses

Statistical analysis was performed by STATISTICA ver.14 (TIBCO Software Inc., Palo Alto, CA, USA) and GraphPad Prism v.6 (GraphPad Software Inc., San Diego, CA, USA). The normality of data distribution was tested by the Kolmogorov–Smirnov test. Data were analyzed by descriptive analysis (means ± SD in case of normal distribution, or medians (lower quartile–upper quartile) in the case of non-normal distribution). Comparisons between groups were performed by Student’s unpaired t-test in case of normally distributed variables and by the Mann-Whitney U-test in case of variables with non-normal distribution. Pearson linear regression was assessed between continuous variables. Backward stepwise multiple regression analyses were performed to determine the variables that best predicted fetuin-A and RBP4 concentrations. We performed statistical power analysis using the calculator of SPH Analytics (SPH Analytics LTD., Alpharetta, GA, USA) to validate the difference of serum fetuin-A and RBP4 levels in obese (group 1) and control individuals (group 2). The statistical power was above 0.8 both on fetuin-A (0.81) and RBP4 (0.92), respectively. Results were considered to be significant at the level of *p* < 0.05.

### 2.7. Results

Anthropometric and routine laboratory parameters are presented on Table 1. Obese patients had markedly higher BMI and waist circumference compared to lean controls. HsCRP, LDL-C, triglyceride, and liver enzyme levels were significantly higher, while HDL-C and ApoAI concentrations were significantly lower in enrolled obese patients compared to controls. These parameters—except for hsCRP—remained in the normal reference range. HDL-associated antioxidant PON1 enzyme activities were found to be slightly lower in obese patients compared to lean controls. Carbohydrate parameters, fasting glucose, and HbA1C levels were slightly elevated in obese patients compared to healthy, lean participants, but they still stayed in the normal range. The obese patients involved in this study had neither diabetes nor abnormal glucose tolerance on the basis of OGTT results. Among obese patients, the ratio of hypertension was 59%, while 10% of patients suffered from ischemic heart disease.

Absolute and relative amounts of distributed lipoprotein subfractions are shown in Table 2. Absolute and relative amounts of VLDL, large LDL, and small-dense LDL subfractions were significantly higher, while the percentage and concentration of IDL subfraction along with mean LDL size were significantly lower in obese than lean participants. During HDL subfraction analysis, we detected a shift toward the small-sized HDL particles in obese individuals (Table 2): relative and absolute amounts of the large HDL subfraction were significantly lower, while the percentage and concentration of the small HDL subfraction were significantly higher in obesity. 

**Figure 1:**
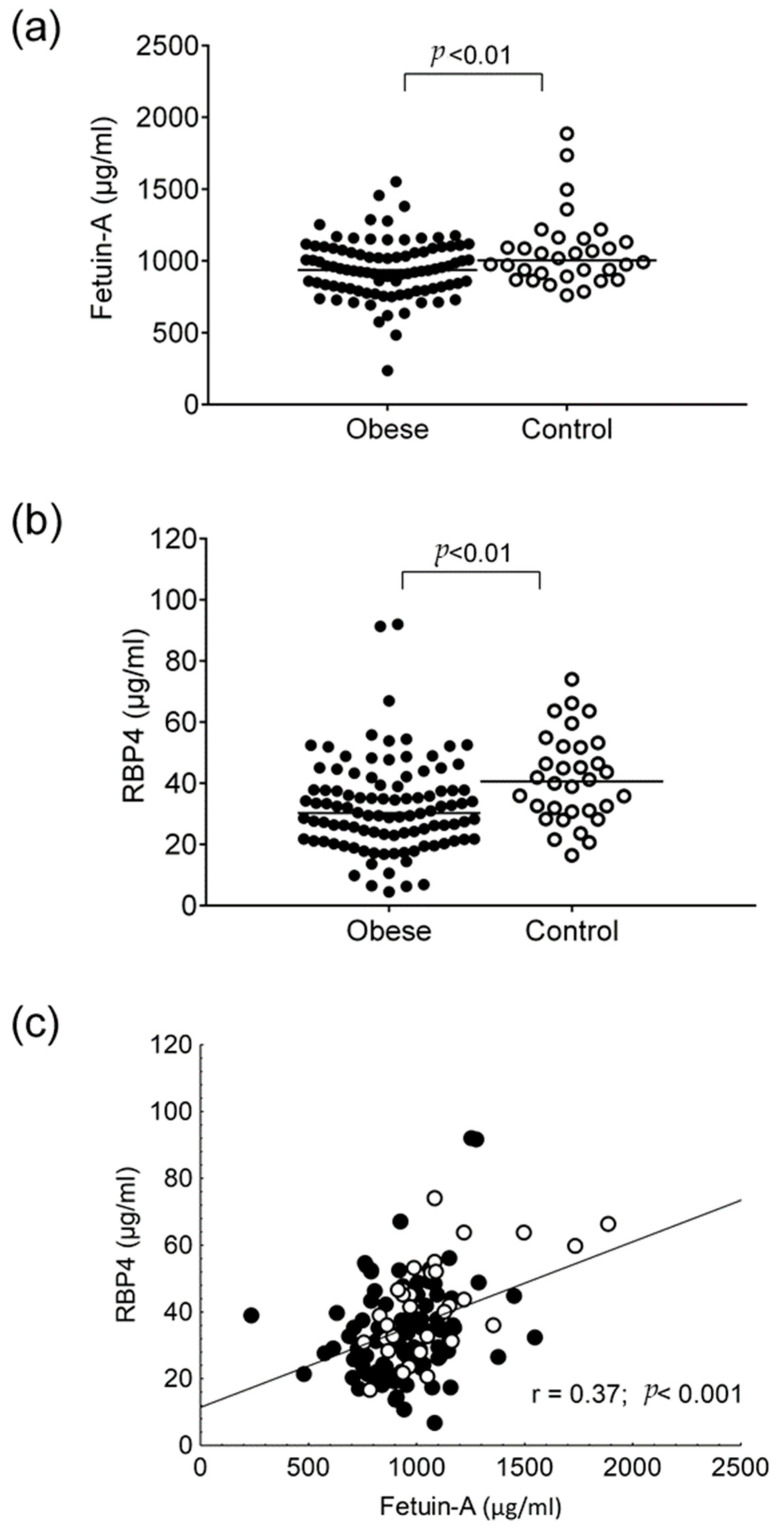
(**a**) Serum concentrations of fetuin-A and (**b**) retinol-binding protein 4 (RBP4); and (**c**) correlation with each other in obese non-diabetic (●) and lean participants (○).

Serum fetuin-A and RBP4 concentrations were found to be significantly lower in obese patients compared to lean controls (fetuin-A: 944.6 ± 201.9 vs. 1068.8 ± 253.3 μg/mL, *p* < 0.01; RBP4: 32.3 ± 15.0 vs. 41.4 ± 14.4 μg/mL, *p* < 0.01; respectively) (Figure 1a,b). We did not detect gender differences between fetuin-A and RBP4 levels (*p* = 0.76 and *p* = 0.97, respectively). There was a positive correlation between fetuin-A and RBP4 in overall participants (r = 0.37; *p* < 0.001) (Figure 1c). It must be noted that investigating the correlation between fetuin-A and RBP4 concentrations in each group separately, data remained statistically significant (obese: r = 0.23; r = 0.03 and lean: r = 0.59; *p* < 0.001).

**Figure 2:**
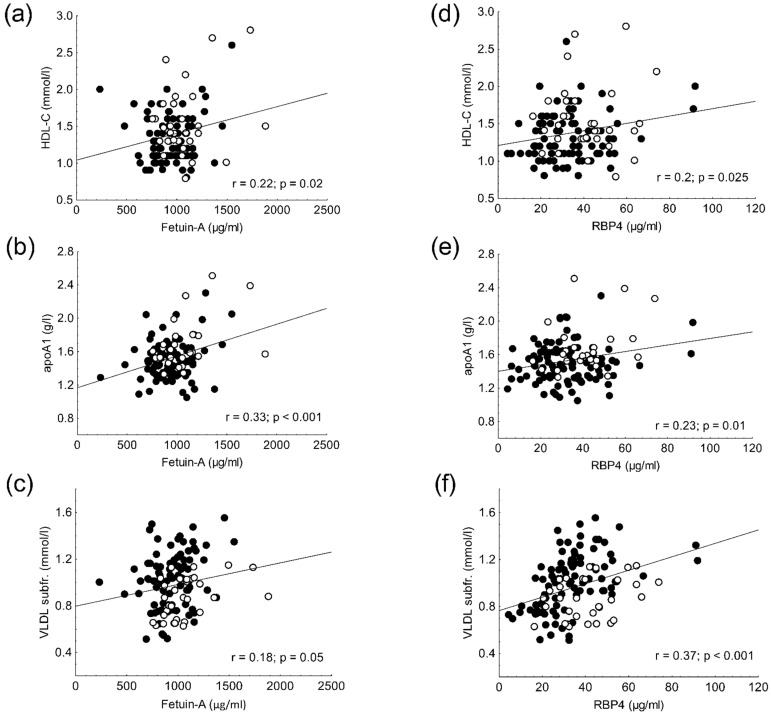
Correlations between serum fetuin-A and (**a**) high-density lipoprotein cholesterol (HDL-C); and (**b**) apolipoprotein AI (apoAI); and (**c**) very-low density lipoprotein (VLDL) subfraction levels. Correlations between serum retinol-binding protein 4 (RBP4) and (**d**) HDL-C; and (**e**) apoAI and (**f**) VLDL subfraction levels in obese non-diabetic (●) and lean participants (○).

**Figure 3:**
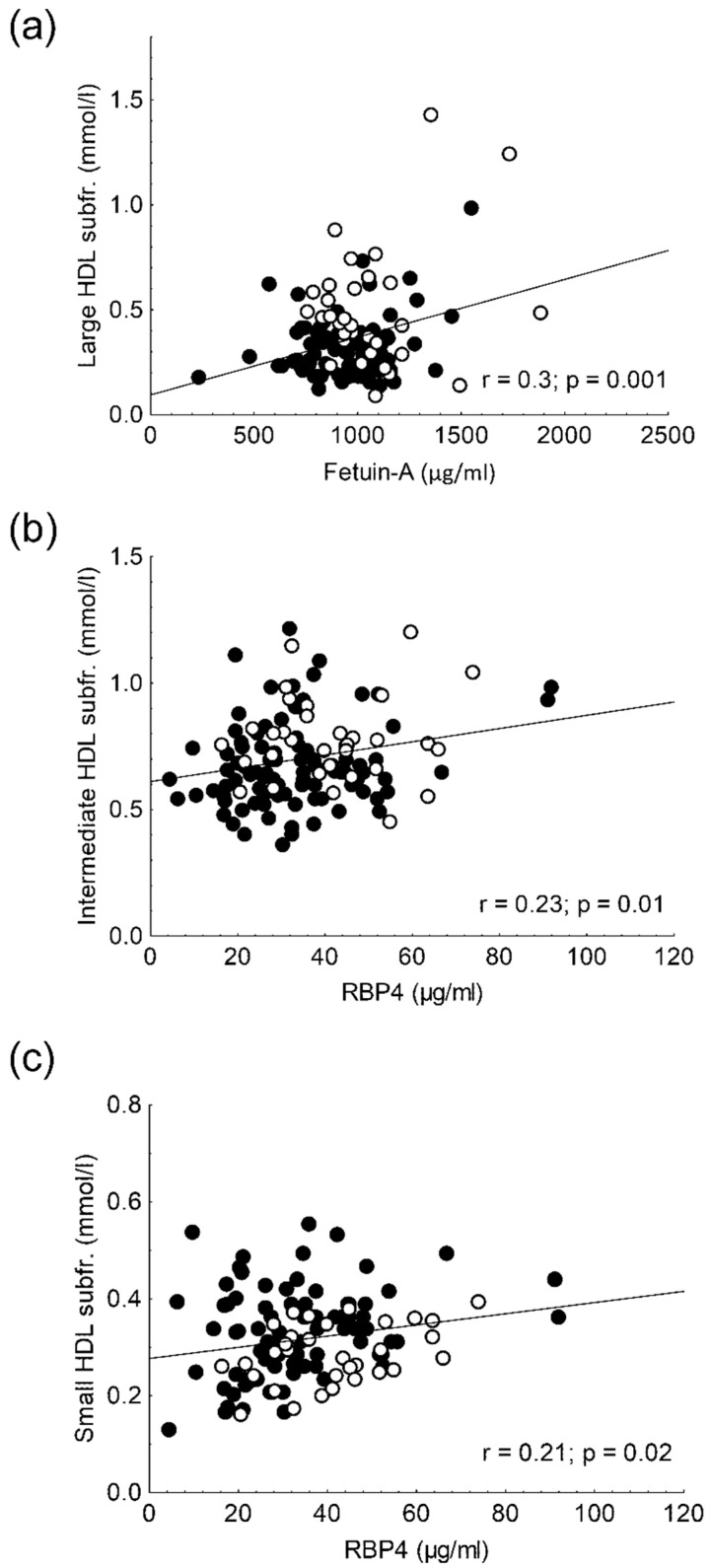
(**a**) Correlation between serum fetuin-A and large high-density lipoprotein (HDL) subfraction levels; correlations between serum retinol-binding protein 4 (RBP4) and (**b**) intermediate HDL subfraction and (**c**) small HDL subfraction levels in obese non-diabetic (●) and lean participants (○).

Circulating fetuin-A correlated negatively with BMI (r = −0.24; *p* = 0.01) and waist circumference (r = −0.31; *p* < 0.01), and it correlated positively with total cholesterol (r = 0.28; *p* < 0.01) in all participants (data not shown). Additionally, fetuin-A showed a positive correlation with HDL-C (r = 0.22; *p* = 0.02), apoA1 (r = 0.33; *p* < 0.001), and VLDL subfraction levels (r = 0.18; *p* = 0.05) in two groups (Figure 2a–c). In addition, there was a positive correlation between fetuin-A and large HDL subfraction levels (r = 0.3; *p* = 0.001) in overall participants (Figure 3a). Carbohydrate parameters (fasting glucose, fasting insulin, HOMA-IR, C-peptide) did not show correlation with serum fetuin-A concentration. 

Circulating RBP4 levels negatively correlated with BMI (r = −0.22; *p* = 0.02), waist circumference (r = −0.23; *p* = 0.02), and fasting glucose (r = −0.31; *p* = 0.003) in all the enrolled subjects, while there was a positive correlation between RBP4 and C-peptide levels (r = 0.36; *p* < 0.01) in obese non-diabetic patients (data not shown). We also found positive correlations between RBP4 level and HDL-C (r = 0.2; *p* = 0.025), apoAI (r = 0.23; *p* = 0.01), VLDL subfraction (r = 0.37; *p* < 0.001), and intermediate HDL subfraction (r = 0.23; r = 0.01) as well as small HDL subfraction levels (r = 0.21; *p* = 0.02) in overall participants (Figure 2d–f and Figure 3b,c).

Age and HDL-linked PON1 enzyme activities did not show correlations with serum fetuin-A and RBP4 concentrations (data not shown).

Backward stepwise multiple regression analysis (Model 1) demonstrated that serum fetuin-A level turned out to be best predicted by RBP4 (ß = 0.334, *p* < 0.001) and large HDL subfraction (ß = 0.364, *p* < 0.001), when the model included BMI, waist circumference, RBP4, HDL-C, total cholesterol, apoAI, VLDL subfraction, and large HDL subfraction level. Moreover, we carried out a backward stepwise multiple regression analysis with RBP4 level as a dependent variable. Model 2 included BMI, waist circumference, fetuin-A, C-peptide, HDL-C, apoAI, VLDL subfraction, intermediate HDL subfraction, and small HDL subfraction levels. Based on this analysis, RBP4 level was predicted by VLDL subfraction (ß = 0.477, *p* < 0.001).

## 3. Discussion

To the best of our knowledge, this is the first complex examination of the correlations of serum fetuin-A and RBP4 concentrations in relation to lipoprotein subfractions in obese non-diabetic patients and healthy normal weight controls. Despite the extremely high body mass index, these obese patients had normal physiological lipid and carbohydrate values. Surprisingly, significantly lower fetuin-A and RBP4 levels were found in morbid obese non-diabetic subjects compared to lean controls. Fetuin-A and RBP4 levels correlated with each other. Multiple regression analysis showed that serum fetuin-A concentration is best predicted by RBP4 and large HDL subfraction. In model 2, VLDL subfraction was the independent predictor of serum RBP4 level. Our results may indicate a potential role of fetuin-A and RBP4 in impaired lipoprotein metabolism in obesity. In obese non-diabetic subjects, low grade inflammation, obesity-associated dyslipidemia, and moderately impaired carbohydrate parameters were observed; however, with the exception of hsCRP, these parameters remained constant in the physiological reference range. During LDL subfraction analysis, a higher percentage and concentration of VLDL, large LDL, and small-dense LDL subfraction, as well as lower percentage and level of IDL subfraction and mean LDL size were found in obese non-diabetic patients compared to lean controls. These data support the presence of atherogenic lipid profile in obesity [20]. Additionally, corroborating the previous results [21,22], a significantly higher percentage and amount of small-sized HDL subfraction were detected in obese individuals.

To date, data are highly contradictory about the direct association of fetuin-A and RBP4 molecules to HDL particles. Moreover, data are lacking about the relationship between impaired circulating fetuin-A and RBP4 production and the markers of obesity-related dyslipidemia (i.e., small HDL, mean LDL size etc.) in a special morbidly obese but non-diabetic population.

We found unexpectedly lower fetuin-A concentration in obese subjects compared to lean participants. Conflicted findings were described on the level of fetuin-A in obesity and diabetes in previous reports. Recent studies demonstrated elevated serum fetuin-A levels both in obese and overweight adults and children [23,24]. In addition, significantly higher fetuin-A levels were found in type 2 diabetic patients compared to the controls [4]. At the same time, some authors indicated that fetuin-A concentration did not differ in diabetic and non-diabetic obese subjects, and significantly lower fetuin-A level was observed in the metabolically healthy subgroup [25]. Our data on morbidly obese, still relatively healthy, non-diabetic patients support this finding. However, it must be noted that anti-diabetic and lipid-lowering medications (e.g., metformin and statins) can also reduce circulating fetuin-A level [26,27]. As summarized in Table 1, 12% and 14% of enrolled obese participants received metformin or statin treatment, respectively, which could contribute to lower fetuin-A level; however, further examinations are needed to clarify this phenomenon. 

Alongside the involvement of insulin resistance, fetuin-A is associated with dyslipidemia and the components of metabolic syndrome [28]. In this study, serum fetuin-A positively correlated with total cholesterol and VLDL subfraction levels in obese non-diabetic patients and lean controls. These findings are in line with Liu et al. [29], who examined the link between fetuin-A and the distribution of lipoprotein subfractions—performed by vertical auto profile methodology (VAP)—along with fetuin-A in obese patients with obstructive sleep apnea. Similar to another study [24], we described a positive correlation between fetuin-A and HDL-C levels; to our best knowledge, this is the first report about correlation between circulating fetuin-A and apoAI as well as large HDL subfraction levels. Data are highly contradictory about the link between fetuin-A and HDL [6]. Circulating fetuin-A inhibits the activity of insulin receptors mediating phosphatildylinositide 3-kinase (PI3K) and Akt signaling pathways; it also inhibits GLUT4 translocation, which could lead to lower HDL-C level. Meanwhile, HDL can promote glucose uptake in the adipocytes and glycogen synthesis through enhancing GLUT4 involving PI3K/Akt via AMPK signaling pathways. Previously, fetuin-A was described as one of the HDL-associated proteins in proteomic analyses [16], but its affinity to various HDL subclasses is not clarified. A positive correlation between the levels of fetuin-A and large HDL subfraction may indicate that fetuin-A is associated to large HDL particles, and its glycosylation, reported previously [30], may promote its enhanced clearance from the circulation, leading to a lower level of large HDL subfraction. Further studies on HDL function are needed to evaluate this hypothesis. 

In line with fetuin-A, significantly lower RBP4 concentration was found in obese non-diabetic participants compared to lean controls. Numerous studies indicated a pathological level of RBP4 in obesity and diabetes; however, the results are partially contradictory. A previous study demonstrated a U-shaped association between RBP4 concentration and risk of incident of type 2 diabetes: <31 μg/mL and >55 μg/mL RBP4 levels were significantly associated with increased risk of incident of type 2 diabetes in middle-aged prediabetic participants [11]. These results can support our recent data. However, it is important to note that the choice of the assay employed (ELISA, EIA, quantitative Western blotting) in the measurement of circulating RBP4 concentration might be decisive [10]. 

Growing evidence about the role of RBP4 in lipid metabolism and atherosclerosis is well known, but the data are not clearly defined; also, both atherogenic and cardioprotective effects of RBP4 have been described. Previously, positive correlations were shown between RBP4 and triglyceride, total cholesterol, and LDL-C levels in type 2 diabetic patients [31]. Additionally, RBP4 levels are related to large VLDL and small-dense LDL subfractions in subjects with and without diabetes [32]; mean LDL size in morbid obese patients was noticed as well [33]. However, other studies did not confirm these results [34,35]. In this study, we did not observe correlations between RBP4 and total cholesterol, triglyceride, LDL-C levels, and mean LDL size, but there was a positive correlation between RBP4 and VLDL subfraction levels in obese and lean non-diabetic subjects. 

Interestingly, serum RBP4 concentration correlated positively with HDL-C, apoAI, intermediate HDL, and small HDL subfraction levels in obese and lean non-diabetic subjects in our study. Neither fetuin-A nor RBP4 associate with PON1 enzyme activities. Proteomic analyses on HDL described an extended list of HDL-associated proteins. In addition to the widely known HDL-linked structural and enzymatic proteins such as apolipoprotein As, Cs, Es, L, M, PON1, myeloperoxidase, serum amyloid, etc.; other small amounts of proteins could associate with HDL particles, including fetuin-A (α2-HS-glycoprotein) or RBP4. In case of an unfavorable oxidative milieu such as diabetes or heart disease, fetuin-A can be significantly enriched in HDL particles, leading to impaired HDL proteome composition via N-glycosylation and sialylation [30,36]. In patients with chronic kidney disease, almost a two-fold abundance of RBP4 was associated with lower estimated glomerular filtration rate (eGFR) in HDL particles [16]. However, some authors indicated that the data of HDL particle proteome composition are highly variable and strongly depend on the HDL isolation and purification method [17]. These precise results of proteomic analyses could verify our results; however, further examinations are necessary to clarify these findings in obesity.

Our study aimed at better understanding the regulation of lipid metabolism in the obese, non-diabetic patient population to define the effect of obesity on lipid parameters without the concomitant effect of disturbed carbohydrate metabolism. This type of information might be useful as a prognostic factor for elevated cardiovascular risk in obesity and may help to define the therapeutic strategies in obesity and to find novel treatment options to alter lipoprotein levels and to reduce the risk of atherogenesis in obese subjects.

Some limitations must be mentioned. A larger number of obese and control participants and the enrolment of patients with carbohydrate disturbances may enhance the statistical power. Furthermore, it must be noted that investigating correlations in each group separately, only correlation between fetuin-A and apoA1 and correlation between fetuin-A and VLDL subfraction remained statistically significant both in obese and control group. However, despite the relatively small number of participants, our findings may underline the potential role of fetuin-A and RBP4 in impaired lipoprotein metabolism in obese non-diabetic and lean non-diabetic patients. Investigation of structural and qualitative properties of HDL particles may provide further detailed information about impaired HDL function in obesity.

## 4. Conclusions

In this study, unexpectedly lower serum fetuin-A and RBP4 levels were observed in morbid obese non-diabetic patients compared to lean non-diabetic subjects. Fetuin-A and RBP4 levels correlated with each other and also showed correlations with lipoprotein subfractions. Our results may highlight the potential role of these proteins in the obesity-associated lipoprotein disturbances. These findings might serve information about fetuin-A and RBP4 as a prognostic factor for elevated cardiovascular risk in obesity and may help to define the therapeutic strategies and to find novel treatment options to alter lipoprotein levels and to reduce the risk of atherogenesis in obese subjects. Further studies on the HDL proteome and the enrolment of patients with insulin resistance or diabetes might complete our findings.

## Figures and Tables

**Table 1:** Anthropometric and routine laboratory parameters of enrolled participants. Data are presented as mean ± standard deviation or median (upper-lower quartiles).

	Obese	Control	*p*-Value
Number of subjects (*n*)	100	32	
Female (*n*, %)	83 (83)	27 (84)	0.8
Male (*n*, %)	17 (17)	5 (16)	0.8
Age (years)	44.7 ± 12.5	41.8 ± 6	0.2
Body mass index (kg/m^2^)	42.5 ± 8.1	24.5 ± 2.5	<0.001
Waist circumference (cm)	122.3 ± 16.7	83.6 ± 9.3	<0.001
**Medications**			
ACEI/ARB (*n*, %)	49 (49)	0 (0)	
CCB (*n*, %)	22 (22)	0 (0)	
Statin (*n*, %)	14 (14)	0 (0)	
Metformin (*n*, %)	12 (12)	0 (0)	
Diuretics (*n*, %)	33 (33)	0 (0)	
**Laboratory parameters**			
C-reactive protein (mg/L)	8.1 (3.5–15.7)	1.6 (0.6–2.9)	<0.001
Fasting glucose (mmol/L)	5.5 ± 0.6	4.8 ± 0.5	<0.001
OGTT 120 min (mmol/L)	6.5 ± 1.8	na	
Fasting insulin (mU/L)	17.6 ± 9.5	na	
HOMA-IR	3.6 (2.5–5.5)	na	
C-peptide (pmol/L)	1110 (775–1394)	na	
Fructoseamine (μmol/L)	224.5 ± 28.2	229 ± 11.7	0.4
Haemoglobin A1C (%)	5.6 (5.2–6.1)	5 (4.8–5.3)	<0.001
GFR (mL/min/1.73 m^2^)	90 (90–90)	90 (90–90)	0.2
Urea (mmol/L)	5.1 ± 1.6	4.6 ± 1.3	0.2
Creatinine (µmol/L)	66.3 ± 17.5	68.3 ± 14.5	0.6
Cholesterol (mmol/L)	5.0 ± 0.8	5.1 ± 0.8	0.7
HDL-C (mmol/L)	1.3 ± 0.3	1.6 ± 0.5	0.002
LDL-C (mmol/L)	3.2 ± 0.7	2.9 ± 0.5	0.04
Triglyceride (mmol/L)	1.45 (1.1–1.95)	1 (0.7–1.4)	<0.001
Apolipoprotein AI (g/L)	1.48 ± 0.24	1.68 ± 0.3	0.003
Apolipoprotein B (g/L)	0.87 ± 0.2	0.94 ± 0.2	0.1
Thyroid stimulating hormone (mU/L)	1.95 (1.48–2.67)	1.94 (1.26–2.11)	0.1
AST (U/L)	20 (17–27)	18 (16.5–21)	0.02
GGT (U/L)	28 (19–43)	19 (16–28.5)	0.01
ALT (U/L)	25 (18–32)	17 (12.5–23.5)	<0.001
PON1 paraoxonase activity (U/L)	56.7 (37.3–139.2)	83 (47.9–167.4)	0.04
PON1 salt stimulated paraoxonase activity (U/L)	149.8 (105.1–293.3)	169.4 (97.3–297.4)	0.9
PON1 arylesterase activity (U/L)	124.3 ± 22.1	135.3 ± 36.8	0.04

Abbreviations: ACEI/ARB, Angiotensin-converting enzyme inhibitors/Angiotensin receptor blockers; ALT, alanine transaminase; AST, aspartate transaminase; CCB, calcium channel blockers; GFR, glomerular filtration rate; GGT, gamma-glutamyl transferase; HDL, high-density lipoprotein; HOMA-IR, homeostasis model assessment-insulin resistance; LDL, low-density lipoprotein; na, not available; OGTT, oral glucose tolerance test; PON1, paraoxonase 1.

**Table 2:** Distribution of lipoprotein subfractions in non-diabetic obese and lean control subjects. Data are presented as mean ± standard deviation or median (upper–lower quartiles).

	Obese	Control	*p*-Value
**LDL Subfraction Analysis**			
VLDL subfraction (%)	19.9 ± 4.3	17.1 ± 2.3	<0.001
IDL subfraction (%)	25.0 ± 3.9	29.6 ± 5.0	<0.001
Large LDL subfraction (%)	28.0 ± 4.7	21.1 ± 5.8	<0.001
Small-dense LDL subfraction (%)	1.3 (0.0–2.4)	0.5 (0.0–0.85)	<0.01
VLDL subfraction (mmol/L)	1.004 ± 0.243	0.866 ± 0.172	<0.01
IDL subfraction (mmol/L)	1.242 ± 0.296	1.508 ± 0.373	<0.001
Large LDL subfraction (mmol/L)	1.417 ± 0.359	1.074 ± 0.351	<0.001
Small-dense LDL subfraction (mmol/L)	0.069 (0.0–0.129)	0.028 (0.0–0.046)	<0.01
Mean LDL size (nm)	27.1 (26.8–27.3)	27.35 (27.2–27.5)	<0.001
**HDL subfraction analysis**			
Large HDL subfraction (%)	23.2 ± 7.1	29.8 ± 9.0	<0.001
Intermediate HDL subfraction (%)	51.2 ± 3.8	50.8 ± 4.7	0.6
Small HDL subfraction (%)	25.6 ± 6.7	19.3 ± 5.3	<0.001
Large HDL subfraction (mmol/L)	0.308 ± 0.143	0.495 ± 0.287	<0.001
Intermediate HDL subfraction (mmol/L)	0.679 ± 0.171	0.774 ± 0.169	<0.01
Small HDL subfraction (mmol/L)	0.340 ± 0.108	0.287 ± 0.062	<0.01

Abbreviations: HDL, high-density lipoprotein; IDL, intermediate-density lipoprotein; LDL, low-density lipoprotein; VLDL, very low-density lipoprotein.

## Data Availability

All data generated or analyzed during this study are included in this published article. All data generated or analyzed during the current study are available from the corresponding author on reasonable request.
